# Beta-Aminoisobutyric Acid as a Novel Regulator of Carbohydrate and Lipid Metabolism

**DOI:** 10.3390/nu11030524

**Published:** 2019-02-28

**Authors:** Dmitrii A. Tanianskii, Natalia Jarzebska, Andreas L. Birkenfeld, John F. O’Sullivan, Roman N. Rodionov

**Affiliations:** 1 Department of Biochemistry, Institute of Experimental Medicine, Acad. Pavlov St., 12, 197376 St. Petersburg, Russia; dmitry.athero@gmail.com; 2 Department of Fundamental Medicine and Medical Technology, St.Petersburg State University, 8 liter A, 21st Line V.O., 199034 St. Petersburg, Russia; 3 University Center for Vascular Medicine, Technische Universität Dresden, Fetscherstraße 74, 01307 Dresden, Germany; natalia.jarzebska@uniklinikum-dresden.de; 4 Medical Clinic III, Technische Universität Dresden, Fetscherstraße 74, 01307 Dresden, Germany; andreas.birkenfeld@uniklinikum-dresden.de (A.L.B.); john.osullivan@sydney.edu.au (J.F.O.); 5 Charles Perkins Centre and Heart Research Institute, The University of Sydney, 7 Eliza St, Newtown NSW, Sydney 2042, Australia

**Keywords:** BAIBA, myokines, AGXT2, obesity, metabolic syndrome, insulin resistance, AMPK, PPARs

## Abstract

The prevalence and incidence of metabolic syndrome is reaching pandemic proportions worldwide, thus warranting an intensive search for novel preventive and treatment strategies. Recent studies have identified a number of soluble factors secreted by adipocytes and myocytes (adipo-/myokines), which link sedentary life style, abdominal obesity, and impairments in carbohydrate and lipid metabolism. In this review, we discuss the metabolic roles of the recently discovered myokine β-aminoisobutyric acid (BAIBA), which is produced by skeletal muscle during physical activity. In addition to physical activity, the circulating levels of BAIBA are controlled by the mitochondrial enzyme alanine: glyoxylate aminotransferase 2 (AGXT2), which is primarily expressed in the liver and kidneys. Recent studies have shown that BAIBA can protect from diet-induced obesity in animal models. It induces transition of white adipose tissue to a “beige” phenotype, which induces fatty acids oxidation and increases insulin sensitivity. While the exact mechanisms of BAIBA-induced metabolic effects are still not well understood, we discuss some of the proposed pathways. The reviewed data provide new insights into the connection between physical activity and energy metabolism and suggest that BAIBA might be a potential novel drug for treatment of the metabolic syndrome and its cardiovascular complications.

## 1. Introduction

Manifestations of the metabolic syndrome such as abdominal obesity, dyslipidemia, insulin resistance, and hypertension, remain to be the major risk factors for diabetes and cardiovascular diseases. The mechanisms involved in pathogenesis of metabolic syndrome are still not fully understood. A prevailing hypothesis is that the expansion of abdominal fat mass, particularly in the visceral area, leads to “adiposopathy” and, as a result, to dysregulation of secretion of adipose tissue hormones and cytokines, named “adipokines” [1,2]. Obesity is commonly associated with increased production of adipokines, which induce insulin resistance and lipid disorders, such as tumor necrosis factor alfa (TNFα), resistin, and chemerin [3,4,5,6,7], and reduction of secretion of adipose tissue hormones with favorable effects on lipid and carbohydrate metabolism, such as adiponectin and omentin [8,9,10]. Leptin levels are elevated in obesity, but leptin’s insulin sensitizing and anorexigenic effects are abrogated, due to resistance of target cells to the action of this hormone in this condition [11,12]. Interestingly, some adipokines that have beneficial effects on carbohydrate metabolism, such as visfatin, vaspin, and apelin, are paradoxically upregulated in obesity, possibly to partially compensate for the obesity-induced metabolic abnormalities [13,14,15,16,17].

The more recent hypothesis explains the development of obesity-associated metabolic disturbances through alteration in the production of myokines, or “exercise factors”, by skeletal myocytes [18]. Secretion of these molecules, including interleukin (IL)-6, IL-15, and irisin, is strongly upregulated during aerobic physical exercise [18,19]. Acute 100-fold elevation of plasma IL-6 is potentially involved in improvement of glucose uptake and free fatty acids (FFA) oxidation by muscle cells during muscle contraction [18]. Elevation of IL-15 leads to a decline of fat mass in mice [20], and recently discovered myokine irisin induces “browning” of white adipose tissue (the differentiation of resident progenitor cells in white adipose tissue into morphologically and physiologically distinct brown-like adipocytes) and results in increased energy expenditure, body weight reduction, and improvement of diet-induced insulin resistance in mice [21]. 

More recently, β-aminoisobutyric acid (BAIBA), a non-protein amino acid secreted by skeletal muscles upon regular exercise via peroxisome proliferator-activated receptor gamma coactivator 1-alpha (PGC-1α)-dependent mechanism, has been discovered as a novel endogenous protective myokine regulating adipose tissue browning, improving insulin sensitivity and protecting against a high-fat diet-induced obesity [22,23,24]. The goal of this review is to summarize the current knowledge about BAIBA metabolism and its recently discovered protective biological effects, and to discuss the implications of these recently discovered pathways for prevention and treatment of the metabolic syndrome and its complications. 

## 2. Production and Metabolism of BAIBA 

**Figure 1:**
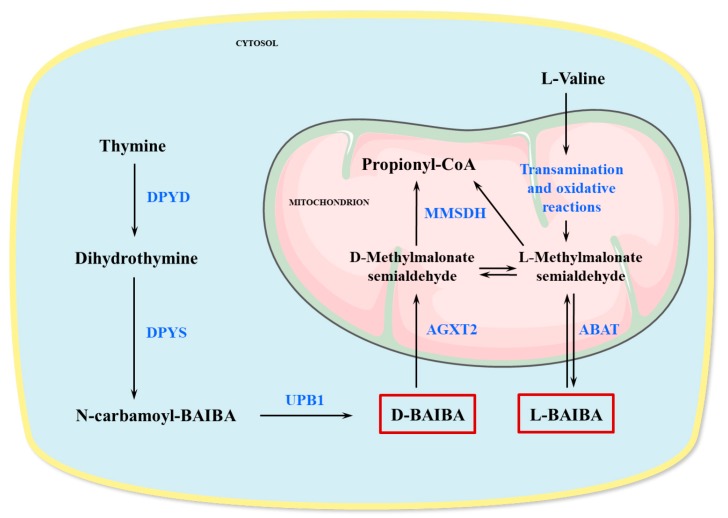
Production and metabolism of β-aminoisobutyric acid (BAIBA). D-BAIBA is produced in cytosole as an intermediate product of thymine degradation, while L-BAIBA comes from mitochondrial reactions of L-valine catabolism. Production and degradation of L-BAIBA is catalyzed by 4-aminobutyrate aminotransaminase (ABAT), a mitochondrial enzyme which is expressed mainly in the brain, kidney, liver, and muscles. D-BAIBA degradation also occurs in mitochondria with the participation of liver’s and kidneys’ enzyme alanine:glyoxylate aminotransferase 2 (AGXT2). The catabolic products of BAIBA enantiomers, L- and D-methylmalonate semialdehydes (L- and D-MMS), enzymatically oxidase with formation of propionyl-CoA. Some amount of L-BAIBA is being converted to D-BAIBA, and vice-versa, through the stereo-isomerization reaction between L- and D-MMS. DPYD—dihydropyrimidine dehydrogenase, DPYS—dihydropyrimidinase, UPB1—β-ureidopropionase, MMSDH—methymalonate semialdehyde dehydrogenase.

BAIBA was originally discovered in human urine in 1951 [25]. There are two enantiomers of BAIBA in biological systems: D-BAIBA (R-BAIBA) and L-BAIBA (S-BAIBA) [26,27]. The literature on the distribution of D-BAIBA and L-BAIBA in plasma, urine, and tissues is contradictory. While most of the studies suggest that D-BAIBA is the main enantiomer of BAIBA in the urine [26,28], some authors report L-BAIBA as the major enantiomer of BAIBA in plasma [26], while others claim that the more prevalent isoform is D-BAIBA [29]. D-BAIBA is produced in the cytosol from thymine in a metabolic pathway involving dihydropyrimidine dehydrogenase (DPYD), dihydropyrimidinase (DPYS), and β-ureidopropionase (UPB1) [30] and is further metabolized in mitochondria by alanine:glyoxylate aminotransferase 2 (AGXT2) to D-methylmalonate semialdehyde (D-MMS) [31] (Figure 1).

AGXT2 is a mitochondrial enzyme that uses pyridoxal phosphate as a cofactor and is present in the liver and also in epithelial cells of the kidney in the loop of Henle [32,33]. This enzyme has broad substrate specificity, the subject of which has recently been reviewed by our group [34]; however, the Km value for D-BAIBA at physiological pH (0.12 mM) is lower than for other amino donors, suggesting that D-BAIBA may be the preferred AGXT2 substrate [31]. 

L-BAIBA is generated from catabolic reactions of branched-chain amino acid L-valine [35,36] (Figure 1). Specifically, L-BAIBA is produced by the mitochondrial enzyme 4-aminobutyrate aminotransferase (ABAT) in the transaminase reaction between the downstream metabolite of L-valine L-methyl-malonyl semialdehyde (L-MMS) and L-glutamate [37,38,39]. ABAT is mainly expressed in the liver, brain, kidneys, muscles, and to a lower extent in the other tissues [37,40]. The optimal pH for this enzyme is 9.1, although it’s also active at lower pH values [38]. Other amino acid substrates for ABAT (pH 8.6) are γ-aminobutyric acid (GABA), β-alanine, δ-aminovaleric acid, but not D-BAIBA or α-amino acids [38]. The reaction of L-BAIBA production by ABAT is thought to be bidirectional, thus the same enzyme can also catalyze the conversion of L-BAIBA to L-MMS [37]. Both L-MMS and the metabolite of D-BAIBA D-MMS can be oxidized by methymalonate semialdehyde dehydrogenase (MMSDH) to propionyl-CoA [36]. Some amount of L-BAIBA is being converted to D-BAIBA, and vice-versa, through the stereo-isomerization reaction between L- and D-MMS [36,41]. The enzyme, which might be responsible for this reaction has not been found yet, while a nonenzymatic mechanism has also been proposed [41]. 

The regulation of systemic levels of D-BAIBA and L-BAIBA is still not entirely understood. It is known that patients with *AGXT2* single nucleotide polymorphisms (SNPs) develop an autosomal recessive metabolic trait hyper-D-β-aminoisobutyric aciduria, which is characterized by elevation of D-BAIBA levels in plasma and urine [42,43]. Interestingly, this trait is presumed to be one of the most common metabolic traits in humans, affecting more than one third of certain Asian populations [44]. Roberts and colleagues reported that BAIBA levels were increased in plasma of mice after exercise-induced activation of PGC-1α, even though the authors did not measure D-BAIBA and L-BAIBA separately in their experiment [22]. Kitase and colleagues showed that production of L-BAIBA is increased during muscle contraction, presumably due to intensive oxidation of L-valine [45]. It is still unknown, whether systemic D-BAIBA levels are also affected by exercise or whether this regulation is only specific for L-BAIBA. 

One of the major limitations in our understanding of the physiological effects of D-BAIBA and L-BAIBA is that most of the supplementation studies in animal models were performed with the D,L-BAIBA racemate, which makes it impossible to determine which of the BAIBA enantiomers were responsible for the observed effects.

## 3. Metabolic Effects of BAIBA

**Figure 2:**
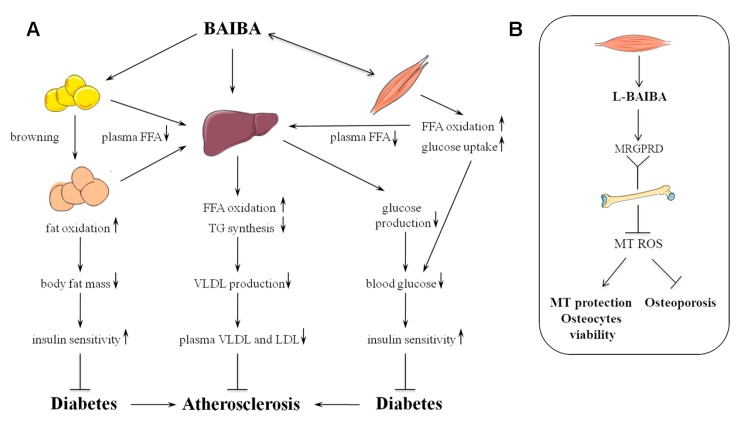
Proposed mechanisms of the biological effects of BAIBA. (**A**) Produced by skeletal myocytes and probably by other cell types, BAIBA regulates lipid and carbohydrate metabolism in fat tissue, liver, and skeletal muscles. BAIBA induces white to brown-like transformation of preadipocytes, which leads to an increase in fatty acids oxidation; it stimulates synthesis and/or activity of free fatty acids (FFA) oxidation enzymes in myocytes and hepatocytes as well. Together these processes lead to a lowering of plasma FFA level with subsequent decline in triglycerides (TG) synthesis and hepatic assembly of very low density lipoproteins (VLDL), the precursors of atherogenic low density lipoproteins (LDL) in the plasma. Decrease in body fat mass induced by adipose tissue “browning”, together with stimulation of skeletal muscles’ glucose uptake and down-regulation of hepatic glucose production enhance insulin sensitivity and reduce risk of diabetes and atherosclerosis. (**B**) L-BAIBA, but not its D-isoform binds to Mas-related G protein-coupled receptor type D (MRGPRD) on osteocytes. L-BAIBA diminishes reactive oxygen species (ROS) production in mitochondria (MT) and protects osteocytes from apoptosis, which results in prevention of bone loss [45].

**Figure 3:**
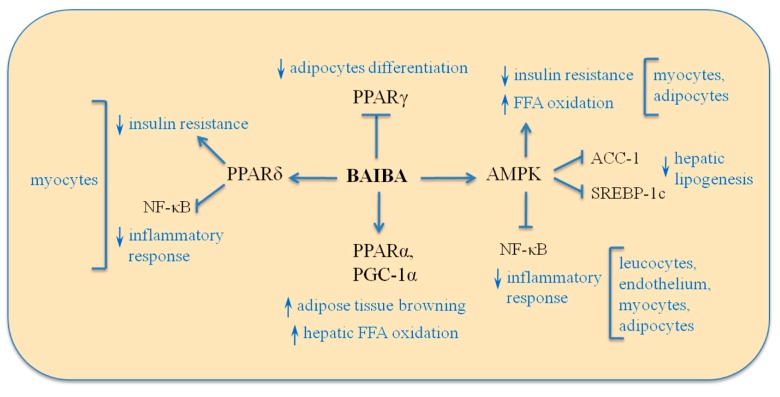
Signaling mediators of metabolic and anti-inflammatory effects of BAIBA. Multiple effects of BAIBA on metabolism and inflammation are mediated by activation of AMP-activated protein kinase (AMPK) and involvement of regulators of gene expression, such as peroxisome proliferator-activated receptors (PPAR)α/δ/γ, PPARγ coactivator 1α (PGC-1α), as well as transcription factors Nuclear factor kappa B (Nf-κB) and Sterol regulatory element-binding protein-1c (SREBP-1c). ACC-1—acetyl-CoA carboxylase, FFA—free fatty acids.

The initial discovery of the metabolic effects of BAIBA was made during mice studies investigating the effects of nucleoside reverse transcriptase inhibitors (NRTIs) on fat metabolism, in which it was shown that thymidine nucleosides and their intermediate product BAIBA, but not the other pyrimidines, increased hepatic FFA β-oxidation, ketone bodies production, and mRNA levels of the rate-limiting β-oxidation enzyme carnitine palmitoyltransferase 1 (CPT-1) in hepatocytes [46]. It was suggested that increased FFA oxidation through BAIBA might have been at least partially responsible for the cases of lipoatrophy of peripheral fat mass in human immunodeficiency virus infected patients receiving thymidine NRTIs [47,48]. Studies in murine models of obesity have shown that chronic treatment (2 weeks to 4 months) with BAIBA leads to a decline in body fat mass [22,23,48], induction of adipose tissue “browning” [22], increasing insulin sensitivity [22,23,24] and FFA oxidation [23,46,48] with lowering [24,49] or neutral [48,49] effects on plasma lipid levels, suggesting that the metabolic effects of BAIBA are not limited to the settings of the NRTI-induced peripheral fat loss. The major effects of BAIBA on lipid and carbohydrate metabolism and its signaling pathways are depicted in Figure 2 and Figure 3.

### 3.1. Adipose Tissue Browning

BAIBA-induced body fat loss was not associated with changes in energy intake [22,23,48] or physical activity in mice [22], however, it correlated with increased aerobic energy expenditure in these animals [22]. These data correspond well with the BAIBA stimulatory effects on FFA oxidation in the liver [22,46] and skeletal muscle cells [23], and on the oxygen consumption by adipose tissue and hepatocytes [22]. Moreover, Roberts et al., 2014 found that BAIBA stimulated differentiation of white adipose tissue preadipocytes to a beige or, so called “brite” (brown in white) phenotype under *in vivo* and *in vitro* conditions. BAIBA induced gene expression of the mitochondrial uncoupling protein UCP-1, mitochondrial biogenesis transcription coactivator PGC-1α, and respiratory chain protein cytochrome c in differentiating adipocytes [22]. These effects were dependent on peroxisome proliferator-activated receptor (PPAR)α, the nuclear receptor, and major transcription activator of FFA oxidation and brown adipose tissue metabolism [22,50]. Increased fatty acids combustion, together with lowering of ATP production efficiency via uncoupling mechanisms, could be an explanation for the decreased body fat accumulation induced by BAIBA.

Adipose tissue browning may also improve plasma lipid profiles and blood glucose levels. Indeed, increased uptake of plasma nutrients such as glucose, triglycerides (TG)-rich very low density lipoproteins (VLDL), and FFA by cold-activated brown adipose tissue in mice was associated with a decline of plasma TG levels and increased insulin sensitivity [51]. The question remains, however, whether browning of white adipose tissue, particularly induced by BAIBA, leads to similar effects on lipid and carbohydrate metabolism, which are observed during activation of the “classic” brown adipocytes and if this explains the observed weight loss.

### 3.2. Lipid Metabolism and Insulin Sensitivity

Interestingly, even though BAIBA treatment in lean mice increased ketogenesis, an indicator of increased hepatic FFA oxidation, it did not alter plasma lipid parameters including FFA, TG, total cholesterol, and phospholipids concentrations [48]. Moreover, the hepatic TG content also did not decrease. One potential explanation for these results could have been an upregulation of lipolysis in adipocytes as a compensation mechanism to overcome decreased FFA levels. Compensatory increase in FFA delivery to hepatocytes may have preserved hepatic TG synthesis and lipoprotein formation after activation of FFA oxidation. Begriche et al., 2008 reported that incubation of mouse adipocytes with 3 mM BAIBA did not affect glycerol production [49], which supports the hypothesis that lipolysis could be an indirect compensatory effect, rather than a direct effect of BAIBA.

BAIBA treatment also had no effect on plasma lipid parameters in mice with high-fat diet-induced obesity or in obese leptin-deficient *ob/ob* mice [48,49], but led to a decline in postprandial levels of TG and fasting cholesterol levels in *ob/+* mice with partial leptin deficiency [49], and reduced fasting FFA, TG, and low density lipoproteins (LDL) cholesterol levels in high-fat diet/low-dose streptozotocin model of type 2 diabetes mellitus in mice [24]. Hypolipidemic effects of BAIBA in the last two models were associated with decreased hepatic lipogenesis and improvements in insulin sensitivity [24,49], while these effects were not observed in *ob/ob* mice [49]. The authors speculated that the observed effects of BAIBA might have been at least partially leptin-dependent, because these effects disappeared in the model of complete leptin deficiency.

Currently, it is not known whether BAIBA has direct effects on hepatic triglyceride synthesis and lipid oxidation. Studies *in vitro* have produced contradictory results, with PPARα-dependent up-regulation of CPT-1 and acyl-CoA oxidase mRNA [22] and no effect on palmitate oxidation in response to treatment of cultured hepatocytes with BAIBA [49]. Interestingly, BAIBA stimulated phosphorylation of AMP-activated protein kinase (AMPK) in human hepatoma HepG2 cells in glucosamine-induced endoplasmic reticulum (ER)-stress settings, but not in the control glucosamine free cells [24]. Phospho-AMPK is the active form of the enzyme AMPK, which phosphorylates and inhibits the key enzyme of *de novo* TG synthesis acetyl-CoA carboxylase and up-regulates CPT-1 activity due to lowering concentration of its inhibitor malonyl-CoA [52]. Moreover, AMPK down-regulates expression of fatty acids synthesis enzymes via phosphorylation and inactivation of the transcription factor SREBP-1c [53]. Animal data suggest that ER stress in hepatocytes is provoked by hepatic excess lipid accumulation and insulin resistance [54]. Activation of AMPK by BAIBA during ER stress may be favorable for lipid metabolism, reducing lipid accumulation and apolipoprotein B (apoB)-containing lipoproteins production in hepatocytes [24]. Future studies will reveal, whether there is any impact of BAIBA on lipogenesis in hepatocytes at the basal conditions or in the settings of ER stress.

Another possible mechanism of hypolipidemic effects of BAIBA observed in some murine models above is modulation of insulin resistance. It is known that improvement of insulin sensitivity has a favorable impact on plasma lipid profile due to insulin-mediated decline in circulating FFA levels and insulin’s blunting effects on hepatic production of apoB-containing lipoproteins [55]. According to Jung and coauthors, 10–30 µM BAIBA decreased palmitate-induced insulin resistance in mouse C2C12 myocytes and lipopolysaccharides (LPS)-induced insulin resistance in mouse 3T3 adipocytes, together with an increase in glucose uptake and activation of FFA oxidation enzymes expression in both cell types [23,56]. These effects were mediated through activation of AMPK [23,56] and were also dependent on PPARδ in the muscle cells [23]. BAIBA also decreased expression of gluconeogenic enzymes and increased phosphorylation of insulin receptor substrate IRS-1, kinase Akt, as well as AMPK in the livers of mice with experimental type 2 diabetes mellitus induced by high-fat feeding and low-dose streptozotocin treatment [24]. Plasma insulin levels in that study were not changed, which suggests that BAIBA increased insulin sensitivity without influence on insulin secretion, but the question remains whether BAIBA has a direct action on insulin signaling in hepatocytes.

### 3.3. Adipokines and Cytokines

There is a possibility that some of the metabolic effects of BAIBA are mediated by other circulating signaling molecules. Begriche and coauthors reported that BAIBA stimulated leptin production in *ob/+* mice with partial leptin deficiency, but not in wild type mice with normal circulating leptin levels [49]. It is well known that leptin plays a pleiotropic role in human and mouse metabolism, including body weight stabilization (through inducing satiety signaling in the brain and upregulating thermogenesis), increasing FFA oxidation and insulin sensitivity, and decreasing hepatic TG production; these effects are partially mediated through AMPK stimulation [12,57]. Interestingly, BAIBA was metabolically active in mice with partial leptin deficiency, but not in wild type mice with normal leptin production or *ob/ob* mice with total depletion of this hormone. These results suggest that BAIBA may affect lipid metabolism and insulin sensitivity via restoration of leptin levels in patients with reduced production of this hormone [49]. It is estimated that about 5–10% of obese individuals are low leptin secretors [58]. The reasons for the selective effects of BAIBA on leptin secretion in *ob/+*, but not in +/+ adipocytes, are not currently known. Leptin production in adipocytes is partially modulated by negative feedback mechanisms through leptin-mediated activation of the sympathetic nervous system [59,60]. Potentially, a local negative feedback regulation of leptin synthesis and/or secretion exists in adipocytes. Therefore, this may explain the limited effect of BAIBA on leptin production in adipocytes with normal leptin content, but the robust one in adipocytes with insufficient leptin synthesis.

Several studies reported that BAIBA can downregulate production of proinflammatory cytokines, which are increased during the chronic low-grade inflammation in adipose tissue seen in obesity [23,56,61,62,63]. Specifically, BAIBA treatment led to declines in plasma levels of TNFα in mice with high-fat diet-induced obesity [23]. Moreover, BAIBA downregulated palmitate-induced IL-6 gene expression in murine C2C12 myocytes [23], decreased LPS-stimulated TNFα production by adipocytes and mononuclears [56,63], and inhibited LPS-induced monocytes adhesion on endothelium [63]. Those effects were mediated by AMPK-dependent inhibition of nuclear factor kappa B and were also dependent on PPARδ in skeletal muscle cells [23,56,63]. Using genetic or chemical cytokines modulation approaches will be crucial for revealing the role of this anti-inflammatory action of BAIBA on adiponectin production in obesity as well as on insulin resistance, lipid metabolism, and atherosclerosis. Interestingly, even though TNFα inhibits production of adiponectin, which is an adipokine, favorably regulating lipid and carbohydrate metabolism [8,64], effects of BAIBA on adiponectin production or its plasma levels could not be demonstrated experimentally [22,48,49]. Future studies will have to address the relative contribution of the effect of BAIBA on the specific cytokines and adipokines towards the overall protective metabolic effects of BAIBA.

### 3.4. Signaling Mechanism

The mechanisms, through which BAIBA acts on cell metabolism, are still obscure. Several membrane receptors for BAIBA were proposed, including G protein-coupled receptors (GPRs), such as Mas-related GPR type D (MRGPRD) [45,65] and orphan receptor GPR41 [22,49] as well as ligand-gated ion channel receptors for glycine and GABA [66,67].

MRGPRD is mainly localized in neurons of dorsal root ganglia, but its expression is also found in the urinary bladder, testis, uterus, arteries, and bone cells [45,68]. L-BAIBA is more potent than D-BAIBA for prevention of age-induced osteocytes death, and this effect is mediated via the MRGPRD receptor, which also binds β-alanine and GABA [45,65]. Specificity of MRGPRD for D-BAIBA as well as stereospecificity of BAIBA enantiomers towards other potential receptors has not been evaluated.

BAIBA may act on adipocytes through activation of GPR41, another G-protein coupled receptor expressed in different cell types, but predominantly in white adipocytes [49,69,70]. This receptor is activated by short chain fatty acids, including propionate, butyrate, pentanoate, and isobutyrate, but not by GABA [70], making it possible that BAIBA also acts as a ligand. Propionate and BAIBA similarly activated leptin secretion by adipocytes [49], and the effect of propionate, like of other small fatty acids, was mediated by GPR41 [70]. Effective concentration at EC50 for this effect of propionate and butyrate was around 200 µM, which is comparable with their concentrations in peripheral blood, 50–100 µM [70]. However, if BAIBA potentially activates these receptors with similar efficiency, it would likely not have a strong effect *in vivo*, since in this case the circulating concentration of BAIBA would be two orders of magnitude lower than its EC50.

There are data suggesting that BAIBA, like other β-amino acids such as β-alanine, taurine, and β-aminobutyrate, binds to and activates glycine receptors [66]. Besides the brain, these receptors are localized in many somatic cells including macrophages, lymphocytes, endotheliocytes, cardiomyocytes, hepatocytes, and renal cells, where they mediate the anti-inflammatory, immunomodulating, anti-ischemic, and cytoprotective effects of glycine [71]. Moreover, glycine intake had some metabolic effects on sucrose-fed animals, stimulating octanoate β-oxidation, reducing plasma FFA and TG, and reducing intraabdominal fat accumulation [72]. However, it seems unlikely that the metabolic effects of BAIBA are mediated through its action on the glycine receptors due to the differences in the agonist concentration and activity between glycine and BAIBA. Plasma concentration of BAIBA (about 2 µM) is about 100-fold lower than the plasma concentration of glycine, which is around 200 µM [73], and BAIBA, like other β-amino acids studied, has a smaller agonist activity for the glycine receptor than its α-amino acid ligand glycine [66].

Finally, β-alanine, taurine and, possibly, BAIBA, also activate the GABA receptors [67]. Like the glycine receptors, the receptors for GABA are expressed not only in the central nervous system, but also outside the brain, e.g., in hepatocytes, endocrine, and immune cells, modulating cell proliferation and immune response [74,75,76]. It is not known whether GABA has a metabolic impact, but there is a probability that BAIBA, the GABA isomer, acts through the peripheral GABA receptors.

In addition to membrane receptor activation, other pathways of BAIBA signaling may include transport of this molecule inside the cell through specific transport proteins such as the taurine transporter (TauT) selective for β-amino acids [77]. Once internalized, BAIBA may activate cytoplasmic or nuclear receptors with subsequent modulation of gene expression, influence on allosteric regulation of metabolic enzymes or undergo conversion into biologically active compounds, e.g., peptides or chemical modifications. Some effects of BAIBA may be mediated through its binding to AGXT2 that results in competitive inhibition of metabolism and leads to concurrent elevation of other substrates of this enzyme such as asymmetric dimethylarginine (ADMA), symmetric dimethylarginine (SDMA) [33], and homoarginine [78], which impact the cardiovascular system and metabolism [79,80,81,82]. Hopefully, future studies will decipher the actual BAIBA signaling mechanism.

## 4. Human Implications

In a large human cohort study (*n* = 2067), plasma levels of BAIBA correlated inversely with plasma concentrations of glucose, insulin, TG, and total cholesterol [22]. These results are in accordance with the positive impacts of BAIBA on adiposity, insulin resistance, and lipid parameters in mice described earlier [22,23,24]. At the same time, these studies do not prove the causality of BAIBA action in human metabolism; one of the approaches to assess causality is to perform genetic investigations. The rs37370 (c.506T>C, Asn102Ser) *AGXT2* SNP has been associated with elevation of circulating BAIBA levels, decreased cholesterol esters plasma content, and, surprisingly, with increased plasma TG levels [83]. The reasons for the contrasting relationship of the analyzed *AGXT2* SNPs and BAIBA concentrations with plasma TG are not known. *AGXT2* knockdown in zebrafish also led to an increased TG/cholesterol ester ratio, but plasma BAIBA levels were unexpectedly decreased [83]. These data hint that AGXT2 affects lipid metabolism independent from BAIBA, possibly via elevation or reduction of the other substrates of this enzyme. However, replication of the findings from Rhee et al., 2013 [83] will still be required in other populations or clinical groups, and the influence of other *AGXT2* SNPs on lipid metabolism and insulin sensitivity will need to be tested. Furthermore, AGXT2 only metabolizes D-BAIBA, thus *AGXT2* polymorphisms’ studies do not address potential metabolic effects of L-BAIBA.

**Figure 4:**
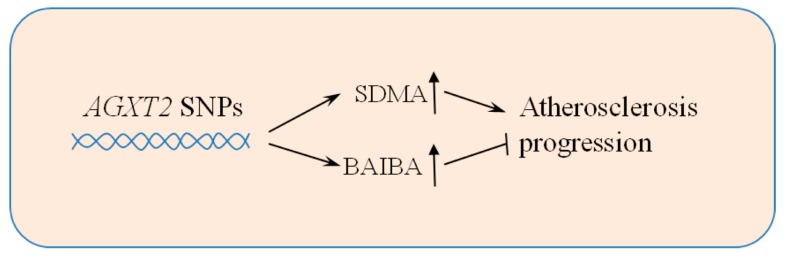
Potentially protective effect of BAIBA on atherosclerosis progression in human subjects with functional *AGXT2* SNPs. *AGXT2* functional SNPs associate with elevation in plasma concentration of both its substrates, SDMA and BAIBA, that oppositely affect atherosclerosis development and cardiovascular outcomes in humans. AGXT2—alanine:glyoxylate aminotransferase 2, SNPs—single nucleotide polymorphisms, SDMA—symmetric dimethylarginine, BAIBA—β-aminoisobutyric acid.

Spitsyn and Afanas’eva showed in 2001 the elevated urine excretion of BAIBA in patients with coronary atherosclerosis [84]. The *AGXT2* C-A-A-A haplotype (rs37370, rs37369, rs180749, rs16899974) in the Japanese population has been positively associated with both BAIBA urine excretion and carotid intima-media thickening [85]. Meanwhile, it is not known whether this haplotype has an impact on other AGXT2 substrates such as ADMA, SDMA [34], as well as homoarginine [78]. Interestingly, elevated plasma levels of ADMA and SDMA are linked to a poor cardiovascular prognosis [79,86], while homoarginine concentration negatively correlates with cardiovascular disease outcomes [80,87]. These observations tell us about the complexity of the influence of *AGXT2* genetic variants on atherosclerosis and vascular biology, as it depends on ratio between different AGXT2 substrates in plasma and in the individual tissues. The impact of *AGXT2* polymorphisms on its biologically active substrates depends not only on the Km of AGXT2 for those substrates, but also on the activity of the alternative metabolic pathways for those compounds. Thus, *AGXT2* SNP rs37369 and, to lesser extent, rs16899974, were associated with elevation of plasma levels of BAIBA and SDMA, while ADMA, arginine, and homoarginine levels were unchanged [43,88]. In spite of influence on SDMA systemic levels, these polymorphisms were not associated with cardiovascular mortality [88,89], although patients with rs16899974 showed an increased incidence of ischemic stroke [90]. These results might be possibly explained by potential protective effects of BAIBA, which may compensate for the negative impact of SDMA on atherosclerosis progression (Figure 4). Future studies could test this possibility by determining, whether BAIBA has any direct protective effects on atherosclerosis.

## 5. Future Directions

One of the major challenges in understanding the physiological and pathophysiological roles of BAIBA is that we are actually dealing with two separate potentially highly biologically active compounds D-BAIBA and L-BAIBA, with different metabolism and different downstream effects. Since most of the available epidemiological and experimental studies do not distinguish between those compounds, it is unclear which of these two compounds is responsible for the observed effects. It is also possible that in some studies opposite effects of D-BAIBA and L-BAIBA cancel each other out, giving misleading results, which are difficult to interpret. One of the major directions of future research will be to systematically determine the relative contribution of D-BAIBA and L-BAIBA to the already discovered effects of BAIBA.

The currently available literature proposes BAIBA as a powerful endogenous regulator of metabolism in humans. BAIBA elevation due to prolonged physical exercise or genetic polymorphisms may be favorable for body fat mass, plasma lipoproteins levels, insulin sensitivity, inflammatory responses, and possibly for the arterial wall. However, the physiological role of BAIBA is still unknown. It may be playing a role as an “exercising factor” similar to IL-6 or irisin [18,19], but the relevance of the effects of these substances during regular exercise for human health and metabolism still needs to be determined. Future studies with muscle-specific downregulation of IL-6, irisin, or BAIBA production, will reveal their significance for metabolic responses during regular exercise. 

It is known that physical activity leads to increased FFA turnover due to activation of lipolysis in adipose tissue and FFA oxidation in muscles; a similar change in FFA metabolism occurs in diabetes. It appears that the metabolic effects of BAIBA occur primarily in conditions of FFA-induced insulin resistance [23,24]. Possible functions of BAIBA in this case may be to overcome the increasing amounts of FFA and insulin resistance in patients with diabetes and abdominal obesity. Therefore, there should be some regulation of BAIBA production under the control of FFA or glucose and insulin. Roberts et al., 2014 revealed that the transcriptional coactivator PGC-1α, which is induced in muscles by physical exercise, stimulates BAIBA production, possibly through expression of enzymes responsible for valine catabolism [22]. However, FFA and insulin both downregulate PGC-1α activity by attenuating its RNA synthesis and phosphorylation [91]. Downregulation of PGC-1α is also associated with mitochondrial dysfunction, which is seen in a diabetic state [92]. Future investigations of the impact of FFA, insulin, hyperglycemia, as well as mitochondrial dysfunction on BAIBA production in muscles and other tissues will help to elucidate whether BAIBA plays a role in overcoming the elevation of plasma FFA and glucose in diabetes. Alternatively, declining production of BAIBA under these conditions can amplify metabolic derangements in diabetic patients. It will also be of interest to check, if BAIBA production is sensitive to the local milieu of cytokines, myokines, and adipokines (e.g., TNFα, IL-6, irisin, adiponectin, and leptin), which also modulate FFA metabolism and insulin sensitivity.

An additional question for future research is the relevance of the metabolic and anti-inflammatory effects of BAIBA [23,56,63] on the development of metabolic syndrome and atherosclerosis in humans. To evaluate whether BAIBA is involved in all these spectra of metabolic disturbances in humans, additional population studies will be required comparing BAIBA plasma concentration and *AGXT2* functional SNPs with severity of atherosclerosis, obesity, diabetes, and metabolic and inflammatory plasma markers. Finally, intriguing questions remain regarding the mechanisms responsible for the metabolic and anti-inflammatory effects of BAIBA. The discovery of its receptor or enzymes modulated by BAIBA will not only unveil the physiological role of this amino acid, but also provide an opportunity to design novel drugs for the treatment of obesity, diabetes, dyslipidemia, and atherosclerosis.

## 6. Conclusions

Interventional studies in mice as well as the results, obtained in cell culture models and human cohorts have demonstrated a novel function of the non-protein amino acid BAIBA—its involvement in regulation of carbohydrate and lipid metabolism. Particularly, BAIBA declines body fat mass, increases insulin sensitivity, favorably affects lipid metabolism, and also decreases inflammatory reactions. It seems that BAIBA preferentially acts in FFA overload conditions, such as physical activity and diabetes. Future research is needed to clarify if dysregulation of BAIBA production and/or its action is involved in pathogenesis of obesity, diabetes, dyslipidemia, and atherosclerosis. The relevance of BAIBA’s specific isomers and recently identified L-BAIBA receptor as well as other potential BAIBA receptors for the signaling effects of this amino acid also need to be determined.
